# Use of Robot-Assisted Gait Training in Pediatric Patients with Cerebral Palsy in an Inpatient Setting—A Randomized Controlled Trial

**DOI:** 10.3390/s22249946

**Published:** 2022-12-16

**Authors:** Fabian Moll, Axel Kessel, Anna Bonetto, Johanna Stresow, Monika Herten, Marcel Dudda, Jens Adermann

**Affiliations:** 1 Klinik für Manuelle Therapie (Hospital for Pain Management), Ostenalle 83, 59071 Hamm, Germany; 2 Department for Trauma-, Hand- and Reconstructive Surgery, University Hospital Essen, University of Duisburg-Essen, Hufelandstraße 55, 45147 Essen, Germany

**Keywords:** gait disorders neurologic, exoskeleton device, cerebral palsy, walking, pediatrics, walk test, hybrid assistive limb, robot-assisted gait training

## Abstract

Robot-assisted gait training (RAGT) provides a task-based support of walking using exoskeletons. Evidence shows moderate, but positive effects in the therapy of patients with cerebral palsy (CP). This study investigates the impact of RAGT on walking speed and gait parameters in pediatric CP patients. Thirty subjects (male = 23; female = 7), with a mean age of 13.0 ± 2.5 (9–17) years, and with spastic CP, were recruited. The intervention group (*n* = 15) underwent six 20-minute RAGT sessions with the Hybrid Assistive Limb (HAL) during an 11-day hospital stay. Additionally, a therapy concept including physiotherapy, physician-performed manual medicine, massage and exercise therapy was provided. The control group (*n* = 15) was treated with the therapy concept only. The outcome was based on a 10-Metre Walking Test (10MWT), 6-Minute Walking Test (6MWT), Gross Motor Function Measure (GMFM-88) and lower extremities passive range of motion. The intervention group achieved a mean increase in walking speed in the 10MWT (self-selected walking speed SSW) of 5.5 s (*p* = 0.378). There were no significant differences between the groups in the 10MWT (max) (*p* = 0.123) and the 6MWT (*p* = 0.8). Changes in the GMFM (total) and in the dimension standing and walking, running and jumping (D + E) showed clinically relevant significant results (*p* = 0.002 and *p* = 0.046). RAGT as a supplement to an inpatient therapy stay appears to have a positive, yet not significant impact on the gait parameters of pediatric CP patients as well as motivating them to practice walking. Further studies with adapted study designs are needed to evaluate different influencing factors.

## 1. Introduction

The therapy and rehabilitation of different pediatric gait disorders of neurological origin present therapists and physicians with a variety of challenges. Cerebral palsy (CP) is one of the most common pediatric neurological disorders with a prevalence of 2.1 cases per 1000 births [1]. The main symptoms of CP are spasticity and dystonia. They are described as a velocity-dependent excessive response to a stretch reflex of the muscle (spasticity) and a sustained or intermittent task-specific muscle- or co-contraction (dystonia) [2]. They result in a reduced stretching ability, increasing resistance to movement and an inhibition of voluntary movement control [3]. The lack of mobility usually creates a plateau in motor development around the age of eight [4,5].

As patients grow up, joint deformation and growing bone length, changing lever and load arms, and weight, present a challenge to many children with CP. Various active and passive treatment [6,7] and care approaches [8] are available. The method for which there is the greatest evidence of success is activity-based therapy delivered through physiotherapy [7,9]. The most extensively studied aspect of therapy is the direct training of motor skills [2]. The care of pediatric patients not only challenges the health care system and thus society, but also the many parents who provide care and therapy for their children. Thus, a structure of caring, therapeutic and supportive caregivers normally forms around the growing patient from infancy onwards, enabling him or her to participate in everyday life. Especially for the parents of children with CP, the question of walking ability is central [3], as the prevalence of depression and anxiety is higher in parents of children with CP compared to those with normally developing children or children with other conditions [10]. Pirpiris et al. [11] report walking ability, along with the ability to stand and perceived pain, as the strongest predictor of satisfaction in children with CP.

Since the early 2000s, research on the use of exoskeletons has increased [12]. Exoskeletons are devices attached externally along the human extremities via one or more joints to assist movement or strength [12]. Bayón et al. [13] reported short-term improvements in strength, mean velocity and gait performance in the Gross Motor Functions Measure (GMFM) after using the CPWalker. In 2018, the rigid exoskeleton Hybrid Assistive Limb (HAL) was used for the first time in pediatric patients with CP [14,15,16,17]. Using interactive biofeedback while walking with the HAL closes the gap between initiated movement and the support of the robot. This makes the HAL unique and motivates the patient to participate more in the therapy. Sczesny-Kaiser et al. [18] report the potential beneficial effects of cortical reorganization in adult patients with a spinal cord injury when they undergo highly repetitive robotic-assisted gait training with the Hybrid Assistive Limb (HAL). Results of case studies and series indicate a significant positive effect of robot-assisted gait training (RAGT) in patients with CP concerning gait speed, mean step length or cadence [19]. However, the quality of the studies is poor to moderate [19] and more studies are needed to evaluate the impacts of RAGT on children with CP. There are no randomized control trials regarding the effect of RAGT with the HAL in pediatric patients with CP compared to a control group.

Van Gorp et al. [20] indicate that functions tend to be lost as patients with CP age. This is mainly based on the argument that adults with CP are less active than their peers without CP. Thus, the question arises which is the most effective way for patients with CP to develop as normally as possible and remain physically active.

The aim of the study is to compare the impact of additional RAGT on pediatric patients with CP using the HAL in an 11-day inpatient hospital stay with a control group receiving only the inpatient therapy without RAGT. We hypothesize that patients with CP benefit from RAGT, with the HAL influencing walking parameters such as walking speed in the 10-metre walking test and gross motor functions.

## 2. Participants and Methods

### 2.1. Study Design

This study was a two arm, single center, randomized, parallel, controlled trial with pre and post assessment approved by the Ethics Committee of the Westphalian Wilhelms University of Münster, Germany (2019-581-f-S). Participants provided written informed consent before data collection. The study was conducted according to the Declaration of Helsinki at the Klinik für Manuelle Therapie, Hamm, Germany.

### 2.2. Recruitment and Inclusion and Exclusion Criteria

Recruitment consecutively took place in the tetraparesis department of the Klinik für Manuelle Therapie, Hamm, Germany, as a randomized sample. The recruitment was carried out by a pediatrician who was blinded to the random allocation.

Due to the availability of the HAL, the recruitment was performed from December 2019 to June 2022. The in- and exclusion criteria (Table 1) were based on the existing literature and the mechanical properties of the HAL.

The study protocol was explained to the subjects and parents by a physiotherapist and a physician and illustrated by videos [23,24].

### 2.3. Randomization and Blinding

Enrolled and consenting patients were randomly assigned to an intervention or a control group similarly in a 1:1 ratio without restriction or stratification using Microsoft Excel for a random sample list. This was conducted by the project manager of the study. Both groups had unmasked staff to screen eligible children and manage the patients (e.g., answer questions concerning the interventions). The participants were recruited from the patient population of the clinic. Blinding could not be guaranteed because the communication of therapy content was discussed in an interdisciplinary team. Likewise, the children could not be forbidden to talk about the robot-assisted therapy with other patients and therapists. The data were evaluated anonymously.

### 2.4. Study Protocol

**Figure 1:**
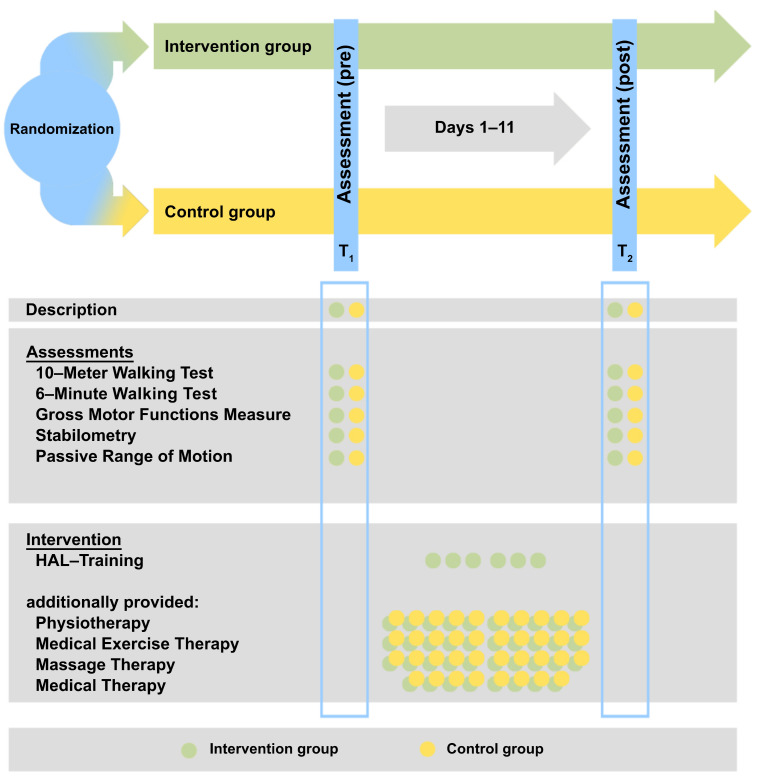
Study protocol (modified from [25]). HAL: Hybrid Assistive Limb.

The study was conducted during a standard inpatient stay at the Klinik für Manuelle Therapie, Hamm, Germany. The intervention time of the study was set by the standard length of the inpatient stay of 11 days. The patients in the intervention group received the inpatient therapy concept and, additionally, six sessions of HAL training. In the control group, the patients only received the inpatient therapy concept (Figure 1).

### 2.5. Hybrid Assistive Limb

The HAL (lower limb type ML-05, Cybderdyne Inc., Tsukuba, Japan) enables individual support of the lower extremities through the use of electromyogram skin surface electrodes and electric motors at the level of the knee and hip joints [26]. Interactive biofeedback is used to graphically display the leg muscles’ derivations. The HAL was used in an inpatient setting requiring a body height of 150–170 cm. It weighs approximately 14 kg. Gait training took place on a treadmill (Kardiomed Mill, Proxomed Medizintechnik GmbH, Alzenau, Germany) using an overhead lift (HM 2815LRC, Handi-Move International, Ninove, Belgium). The HAL was used in cybernetic control mode with individual settings.

### 2.6. Therapy Concept (Intervention)

RAGT with the HAL was carried out by a physiotherapist who was specially trained by Cyberdyne Inc. prior to the performance of the study. Each patient completed six sessions of gait training with the HAL. The training session lasted 90 min and included the actual walking time in the HAL (20 min), time for putting on and taking off the HAL, rests and evaluation of the patients’ skin and well-being before and after.

In addition to the HAL training during the 11-day inpatient hospital stay, the patients also participated in the clinic’s therapy concept consisting of ten sessions of physiotherapy, massage therapy and medical exercise training, and eight 30 min sessions of manual medicine performed by a physician (Table A1). The therapy focused primarily on individual movement restrictions, pain modalities and functional abilities with the goal of achieving patient participation.

### 2.7. Outcome Measures and Statistical Analysis

All clinical assessments were collected at the beginning (day 1 = T1) and end (day 11 = T2) of the inpatient stay. The primary outcome measure was the 10-metre walking test (10MWT) with self-selected walking speed (SSW). As secondary outcome measures, the 10MWT (max), 6-minute walking test (6MWT), Pedoscan and passive range of motion (pROM) of the lower extremities were analyzed. Table 2 gives an overview of the primary and secondary outcome measures.

### 2.8. Statistical Analysis

Descriptive analyses were conducted to summarize socio-demographic, clinical and gross motor characteristics. Normal distributions of the outcome variables were evaluated based on visual observation of histograms and the Shapiro–Wilk-Test. We compared baseline values of walking and gross motor parameters with a multivariate analysis of covariances with repeated measures (r m MANCOVA) “within and between” factors in normal distributed data. The independent-samples *t*-test and equivalent non-parametric tests were used when comparing two groups for continuously and non-normally distributed data. As the therapy concept of the clinic provides a standard therapy concept, and the RAGT was added to this, there was no interim analysis or stopping guidelines planned.

A total of 34 subjects would be sufficient to detect a 0.05 m/s significant increase in walking speed in 10MWT (SSW) with 80% power. The sample size and power were calculated using the G*Power software (version 3.1, Fraul, Erdfelder, Lang, and Buchner, Kiel, Schleswig-Holstein, Germany). The level of statistical significance was set at *p* < 0.05. Confounding variables were selected based on the socio-demographic, clinical and gross motor characteristics. Adverse events were graded according to the Common Terminology Criteria for Adverse Events version 5.0.

The minimal important difference (MCID) in 10MWT in adults with different diagnoses, such as spinal cord injury, stroke and traumatic brain injury, is described with 0.05–0.14 m/s [27,28,29]. No data were found concerning children with CP. MCID in the 6MWT range from 6 to 23 m in patients with CP, showed values of 20–36 m (GMFCS I and II) and 2346 m (GMFCS III) in a subgroup analysis [30]. Gross Motor Functions Measurement (GMFM-88) is indicated to have a total MCID score of 0.13% in children with CP [30]. The statistical analysis was performed using the Statistical Package for Social Sciences (SPSS) (version 28, IBM Corp., Armonk, NY, USA).

## 3. Results

### 3.1. Participants

**Figure 2:**
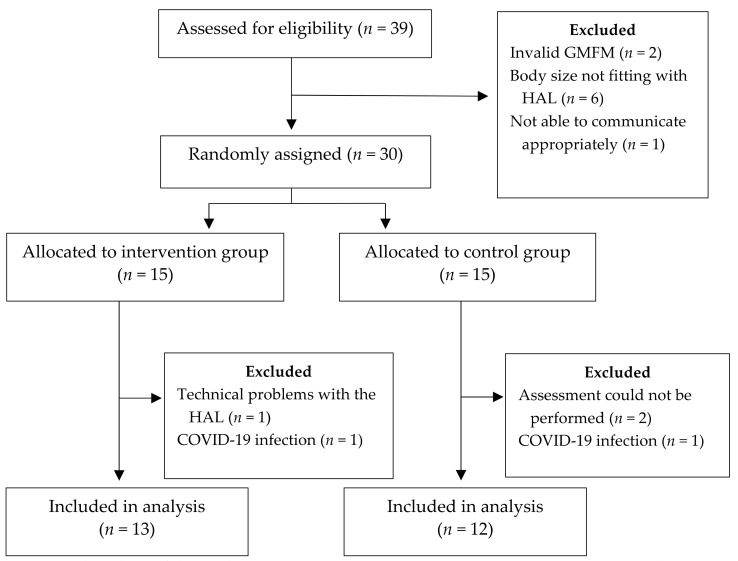
Flowchart of the study. GMFM: Gross Motor Functions Measure; HAL: Hybrid Assistive Limb; COVID-19: Coronavirus disease 2019.

A total of 30 pediatric subjects with CP were recruited from December 2019 to June 2022 (male = 23; female = 7; age: 13.0 ± 2.5 (9–17) years (MV ± SD; range) (Figure 2). All patients were classified according to the Communication Function Classification System as level I. Body-Mass-Index (BMI) was 20.3 ± 3.3 (12.8–25.6) kg/m^2^. Most children (*n* = 28) used a wheelchair as an assistive device in everyday life. Twelve subjects were provided with a retro-walker (also in combination with a wheelchair). Occasionally, other devices were used individually or in combination: 4-point walking sticks (*n* = 9), 1-point walking sticks (*n* = 6), a standing trainer (*n* = 3) and a forearm walker (*n* = 1). Table 3 presents the subject characteristics of the study.

Twenty-five subjects successfully completed the study. Five subjects were dropouts. The reasons for this were technical problems with the HAL (*n* = 1), assessments that could not be performed due to puberty (*n* = 2), and termination of the inpatient stay due to infection of the subject with COVID-19 (*n* = 2). The 25 patients who successfully completed the study were included. Two patients experienced minor adverse events. One subject’s shirt slid up causing a few hours of red skin because of direct skin contact with the lifter’s safety belt. This was gone the next day. One subject had to briefly interrupt the first RAGT session because he was so excited and had very high expectations and was on the edge of syncope. This was solved by rest. No major adverse events were experienced.

The subjects in the intervention group (*n* = 13) all performed six training sessions with the HAL (training time 20 min) in addition to the standard therapy concept. The mean walking distance with the HAL was 851.6 ± 443.1 (231.5–1624.3) meters.

Histograms showed a normal distribution of the data. The MANCOVA was calculated with the 10MWT (SSW), 10MWT (max), 6MWT, GMFM (total), GMFM (D + E) and the different variables of the pedobarography and passive range of motion of the lower extremities. Age, sex, weight, height, and BMI were set as confounding variables. Table 4 gives an overview over the inner-subject contrasts for the functional assessments.

### 3.2. Primary Outcome

**Figure 3:**
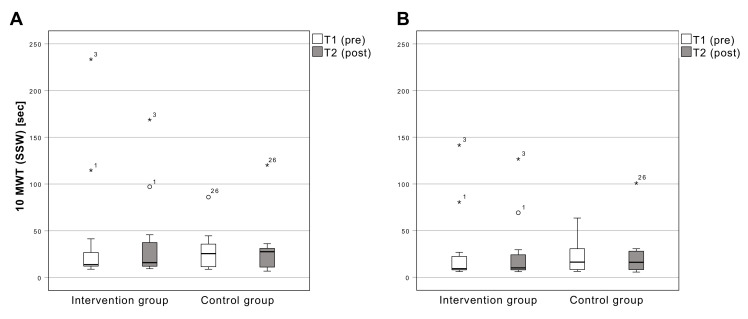
Results of the 10 m walking test with SSW (**A**) and maximum walking speed (**B**). Boxplots: Middle line: median; lower whisker: minimum value; upper whisker: maximum value; circles: Outliers (1.5 times interquartile range); *: extreme outliers (3.0 times interquartile range; numbers: Case numbers); *p* < 0.05; SSW: Self-Selected Walking Speed; T1: measurement pre-intervention; T2: measurement post-intervention; Confidence interval: 95%; *n* = 25.

The time required in the 10MWT (SSW) changed from 41.4 ± 64.3 s (MW ± SD) (T1) to 35.9 ± 46.8 s (T2) in the intervention group and from 28.3 ± 21.8 s (T1) to 30.1 ± 30.2 s (T2) in the control group. There is no significant change (F [1,18] = 0.851; *p* = 0.368) between T1 and T2 (Figure 3).

### 3.3. Secondary Outcome

#### 3.3.1. 10-Meter Walking Test (Max)

The time needed in the 10MWT (max) changed from 28.2 ± 39.4 s (T1) to 25.6 ± 34.9 s (T2) in the intervention group and from 21.7 ± 17.1 s (T1) to 23.0 ± 26.1 s (T2) in the control group. There is no significant change (F [1,18] = 2.62; *p* = 0.123) between T1 and T2 (Figure 3).

#### 3.3.2. 6-Minute Walking Tes

**Figure 4:**
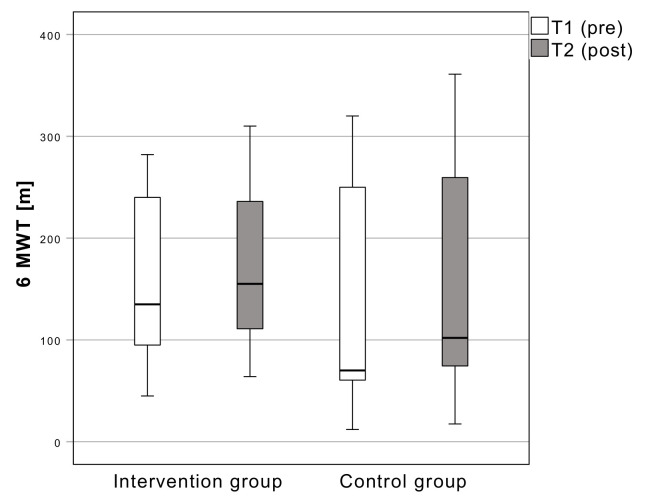
Results of the distance walked in the 6 min walking test. Boxplots: Middle line: median; lower whisker: minimum value; upper whisker: maximum value; circles: Outliers (1.5 times interquartile range); *p* < 0.05; T1: measurement pre-intervention; T2: measurement post-intervention; Confidence interval: 95%; *n* = 25.

The distance walked in six minutes during the 6MWT increased from 156.4 ± 83.8 m (T1) to 168.2 ± 85.3 m (T2) in the intervention group and from 142.6 ± 111.4 m (T1) to 157.3 ± 116.7 m (T2) in the control group. There is no significant change (F [1,18] = 0.07; *p* = 0.800) between T1 and T2 (Figure 4).

#### 3.3.3. Gross Motor Function Measure

**Figure 5:**
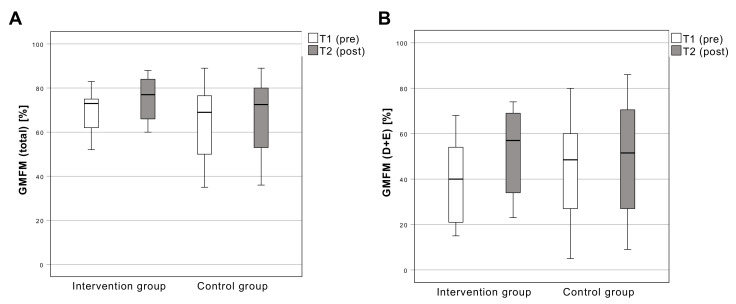
Results of the Gross Motor Functions Measure total (**A**) and Dimension D + E (**B**). Boxplots: Middle line: median; lower whisker: minimum value; upper whisker: maximum value; circles: Outliers (1.5 times interquartile range); *p* < 0.05; T1: measurement pre-intervention; T2: measurement post-intervention; Confidence interval: 95%; *n* = 25.

The GMFM (total) scores increased from 68.5 ± 10.8% (T1) to 75.5 ± 10.1% (T2) in the intervention group and from 64.9 ± 17.7% (T1) to 67.3 ± 18.4% (T2) in the control group. There is a significant change (F [1,18] = 13.12; *p* = 0.002) between T1 and T2 (Figure 1). The GMFM (dimension D + E) scores increased from 40.2 ± 18.8% (T1) to 51.9 ± 18.3% (T2) in the intervention group and from 44.3 ± 23.5% (T1) to 49.4 ± 26.4% (T2) in the control group. There is a significant change (F [1,18] = 4.59; *p* = 0.046) between T1 and T2 (Figure 5).

#### 3.3.4. Pedobarography

**Figure 6:**
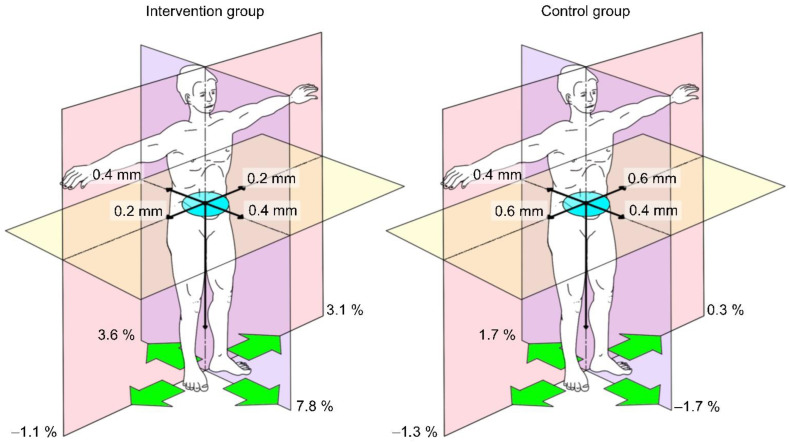
Changes in pedobarography results. Delta from T1 to T2. Pressure distribution (green arrows); anterior-posterior and lateral body center of gravity fluctuation (black arrows); area sway of body center of pressure (blue area); *n* = 16.

For the pedobarography, a saturated model resulted due to the dropouts and two confounding variables (sex and BMI) had to be removed manually. The evaluation via MANCOVA with repeated measures was performed with the covariates age, gender, weight, and height. None of the measurements resulted in significance (Table 5). The performance of the measurements is not valid since most patients found it difficult to stand freely. The results must be considered with reservation (Figure 6, Table 6).

#### 3.3.5. Passive Range of Motion of the Lower Extremities

**Figure 7:**
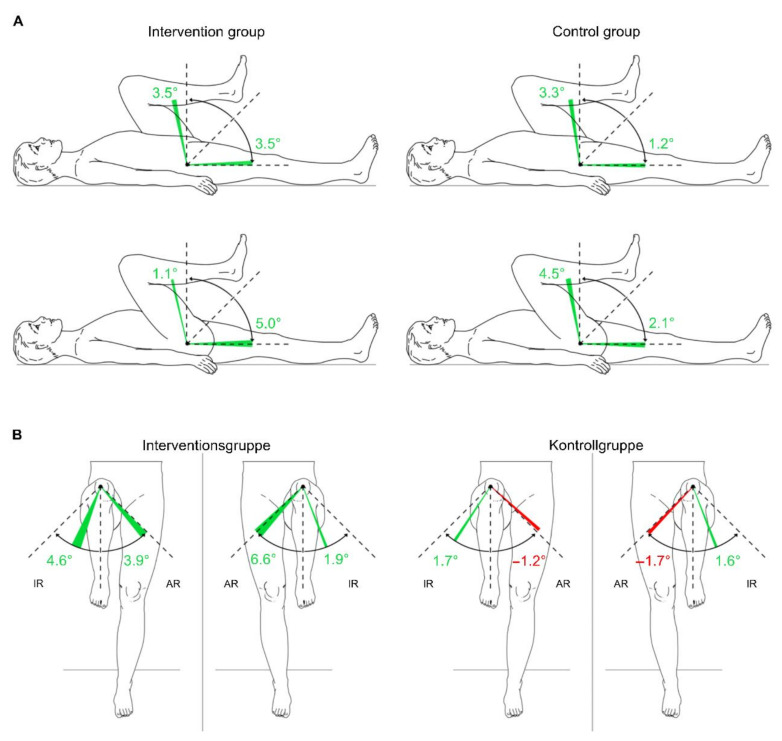
Changes in the passive range of motion of the hip joint in the intervention group and control group from T1 to T2 on average. (**A**): hip joint on sagittal plane; (**B**): hip joint on frontal plane. Green area: positive delta results from T1 to T2; red area: negative delta results from T1 to T2; IR: internal rotation; ER: external rotation; T1: measurement pre-intervention; T2: measurement post-intervention; *n* = 25. Source: authors’ illustration.

**Figure 8:**
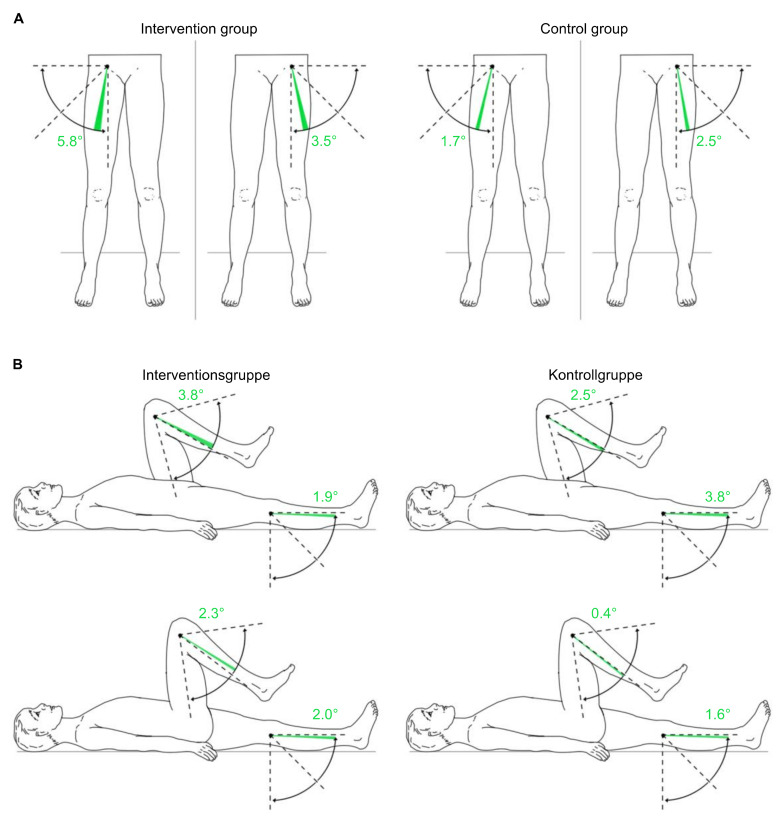
Changes in passive range of motion of the hip and knee joints in the intervention group and control group from T1 to T2 on average. (**A**): hip joint on frontal plane; (**B**): knee joint on sagittal plane. Green area: positive delta results from T1 to T2; red area: negative delta results from T1 to T2; T1: measurement pre-intervention; T2: measurement post-intervention; *n* = 25. Source: author’s illustration.

The MANCOVA of the lower extremities’ pROM displays no systematic difference between the groups at a significance level of 0.05 (Table 7). Only findings for right hip flexion indicate marginally non-significant values, with a change from 105.4 ± 16.8 degrees to 106.5 ± 15.7 degrees in the intervention group and from 101.3 ± 9.1 degrees to 105.8 ± 8.8 degrees in the control group (F [1,18] = 4.423; *p* = 0.050). Most of the pROM changes showed positive results (Table 8, Figure 7 and Figure 8).

## 4. Discussion

HAL gait training can be safely performed and implemented in an inpatient setting. It is also imaginable for other pediatric conditions. This is the first study to compare the functional outcome after an 11-day inpatient therapy stay combined with RAGT in a randomized group of pediatric patients with CP and a control group. In most clinical trials up to now, pre- and post-test designs without a control group and randomization were used [19,31]. Other studies have already reported positive effects on walking parameters after the use of the HAL in children with CP, and this study adds further positive results. However, a comparison with the current evidence is difficult because different exoskeletons and study designs have been used. There is a very high variability in the implementation of RAGT, especially in duration and frequency, and side effects are rarely reported or not at all [31]. To us, there seem to be no risks in implementing the HAL in pediatric patients. Although the benefits of RAGT need to be further investigated quantitatively in pediatric patients with CP, there are beneficial qualitative aspects.

These aspects are: (1) the possibility for the therapist to move freely in the room without stopping the assistance to the patient. (2) For the patient, a setting is created in which he/she can experiment with movements without direct negative feedback. (3) The children also have a much stronger motivation and interest to participate in the therapy, because the robot is a “cool” device.

### 4.1. Limitations

This study shows some limitations. With 25 subjects, the sample size is slightly underpowered. The HAL was only available for a limited time during the research project in the clinic. The current COVID-19 pandemic, which is still prevalent, led to a massive delay in the research project with significantly more patient cancellations. It is also necessary to discuss whether the patients’ performance level may have been affected by the circumstances of the pandemic (social distancing, fewer outpatient therapies, and pulmonary infections). The subjective impression was that patients were less fit at the beginning of the inpatient stay during these two years. Whether this influenced the results positively or negatively could not be answered. Due to the parallel co-interventions, a performance bias should be pointed out. The large number of different interventions will not allow identification of a single effect of RAGT with the HAL on the outcomes. Because children normally learn to walk at an earlier age than the mean age of the participants in this study, which was 13.0 ± 2.5 years, an age bias could affect the results. Because the mechanical properties of the HAL ML05 limited the height of the subjects to 150–170 cm, this was accepted. The smaller version of the HAL may not yet be used in Germany. All assessments in this study evaluate the functional level only. Lefmann et al. [31] also criticize this in their review and point to measures of participation such as the Canadian Occupational Performance Measure (COPM), person and environmental factors.

Pedobarography was difficult to implement in patients with CP due to the lack of standing ability and the orthoses used. This led to nine dropouts and the results must be considered with caution. The passive range of motion of the lower extremities showed slight improvements especially in the extension of the knee and hip in both groups. More functional assessment of the range of motion via video gait analysis would yield a greater quantity of clinically relevant data which could improve the patients’ ability to master the challenges posed by everyday activities. Since functional assessments in patients with neurological diseases can be dependent on the daily form and other environmental factors, and the performance level can vary throughout the day, it must be discussed whether bias could occur here. The assessments were performed in a standardized way and sufficient time was allowed for the execution.

### 4.2. Clinical Relevance

For the primary outcome (10MWT [SSW]), no significant difference could be reported. The mean time in the 10MWT (SSW) decreased by 5.5 s in the intervention group, whereas subjects in the control group took 1.8 s longer comparing T1 and T2. No data on the MCID values in the 10MWT for patients with CP can be found in the literature. For the changes in the 10MWT (max), it can be hypothesized that with a larger sample there might be significant differences between the groups. Regarding the changes in the secondary outcomes, significant differences between groups could be found for the change in GMFM (total) and GMFM (D + E). These are clinically relevant with an absolute change of 7% in GMFM (total) in the intervention group. The statistically significant differences, especially in the dimensions standing, walking, and running, indicate potentially noticeable changes in mobility in everyday life. MCID values for the dimensions D and E could not be found in the literature. The goal of RAGT with the HAL is to practice walking with patients in a contextualized way. This therapy is especially an approach to the fundamental question of all parents of children with CP: When will their child learn to walk? The use of modern technology seems to have motivational and supportive aspects for the child. The HAL can be applied in patients with pediatric CP. Positive influence on walking supporting walking therapy can be seen as well. The use of the exoskeleton gives the therapist the opportunity to explain movement during RAGT. To ensure that the results of this research are also valid in real-life situations, it should be possible to perform the RAGT while walking freely. The walking of curves seems to be especially difficult for many patients with CP and has to be trained. This was not possible when walking on a treadmill. The HAL, as a rigid exoskeleton, allows little room for movement other than in the sagittal plane. Additionally, walking on the treadmill is not the same as walking on normal ground. The children had to look at the floor more often to make sure that their feet were safely meeting the moving treadmill. More studies with qualitative or mixed-method research approaches could provide evidence on whether RAGT with the HAL system is beneficial or more likely to be just a physical strain. The knee and hip extension are of interest in everyday therapeutic practice. They play an important role in the verticalization process and in walking during the stance leg phase. The passive range of motion does not allow direct conclusions to be drawn about changes in the ability to control the joints. However, the increase in the passive range of motion of hip extension of up to 5.1 degrees and knee extension of up to 3.8 degrees in both groups indicates a possible positive non-significant influence of the combined therapy approaches on joint mobility. The extent of using exoskeletons to focus on learning individual movements and functions and integrating them into the gait pattern, as opposed to simply practicing walking, needs to be explored in further research. The significant change in the GMFM may provide an indication that individual functions improve first before they can truly be implemented into the complexity of a more fluid gait pattern.

## 5. Conclusions

Six sessions of RAGT with the HAL system combined with a conventional therapy concept during an 11-day inpatient hospital stay resulted in significant changes in GMFM (total) and GMFM (D + E) values. The use of the HAL in the treatment of pediatric patients with CP seems to have the potential to positively influence walking parameters despite the statistically non-significant changes in the primary outcome. A distinct advantage of the HAL is the active involvement of the patient in the therapy using interactive biofeedback, as it is not possible for a patient in the HAL to just consume the therapy and only pretend to participate. Unfortunately, the very high cost of the HAL means that very few patients can be provided with this form of therapy. Subject characteristics, training parameters, and various neurological conditions of the subjects, as well as various medical conditions and subgroups therefore need further evaluation. Research is needed to evaluate the influence of RAGT with the HAL on younger children with CP at an age when children normally start walking. More standards need to be provided under which RAGT can be implemented. Furthermore, an evaluation of the assessments and the corresponding MCID of the 10MWT, with reference to patients with CP, is necessary to determine more precisely which cut-off values must be exceeded for the results to be clinically relevant. With the background of multiple comorbidities that patients with CP usually present with, it can be hypothesized that long-term RAGT with the HAL and the resulting increased activity may have a positive impact on associated neurological, internal, psychological, and/or gastrointestinal symptoms.

## Figures and Tables

**Table 1:** Inclusion and exclusion criteria (after [16,21,22]).

Inclusion Criteria	Exclusion Criteria
Spastic Cerebral Palsy	Lower limb surgery in the past 6 months
Age 8–18 years	Botulinum toxin therapy in the past 3 months
GMFCS III + II	Use of alternative RAGT in the past 6 months
14 m walking ability	Current fractures or skin lesions
Adequate pain reporting (CFCS level I–III)	Impaired peripheral blood supply
Ability to follow instructions (CFCS level I–III)	Inability to follow instructions
Written informed consent	Height under 150 cm and over 170 cm (for RAGT treatment)

GMFCS: Gross Motor Functions Classification System; RAGT: Robot-assisted gait training; CFCS: Communication Function Classification System.

**Table 2:** Primary and secondary outcome.

Outcome	Assessment	Unit
**Primary**		
Walking speed	10MWT (SSW)	m/s
**Secondary**		
Walking speed	10MWT (max)	m/s
Endurance	6MWT	m/6 min
Gross motor skills	GMFM-88	
	Total	%
	Dimension D (standing) and	%
	E (walking, running, jumping)	%
Joint mobility		
	pROM	Degree (°)
Balance	Pedoscan	mm
	Total a-p COP-Movement	mm
	Total lateral COP-Movement	mm^2^
	Area of sway	%
	Foot pressure (anterior-posterior)	%
	Foot pressure (left-right)	

10MWT: 10-metre walking test; SSW: Self-selected walking speed; max: maximum walking speed; 6MWT: 6-minute walking test; GMFM-88: Gross Motor Function Measure; pROM: Passive range of motion; a-p: anterior-posterior.

**Table 3:** Overview of subject characteristics. GMFCS: Gross Motor Functions Classification System.

Subject	Age	Sex	Height	Weight	BMI	GMFCS	Paresis	Orthosis	Therapy
1	17	F	161	50	19.3	3	Tetra	AFO	PT, OT
2	12	M	140	25	12.8	3	Tetra	AFO	PT, Petö
3	14	M	150	45	20.0	3	Tetra	None	PT, OT, LO
4	11	M	146	38	17.8	3	Tetra	None	PT
5	17	M	165	68	25.0	3	Di	AFO	PT
6	16	F	53	40	17.1	3	Tetra	AFO	PT, OT
7	12	M	156	50	20.6	2	Tetra	AFO	PT
8	14	M	165	52	19.1	2	Tetra	None	PT, OT, HIP
9	11	M	153	42	17.9	3	Tetra	FO	PT, OT
10 ^DO^	11	M	150	43	19.1	2	Tetra	AFO	PT
11 ^DO^	16	F	155	56	23.3	2	Tetra	AFO	PT, OT, LO
12	15	M	160	62	24.2	3	Di	None	PT, OT, LO
13	9	F	125	24	15.4	2	Tetra	FO	PT
14	17	M	170	74	25.6	3	Tetra	None	PT
15	11	M	148	41	18.7	3	Tetra	AFO	PT, OT, HIP, LO
16	12	M	160	62	24.2	2	Di	AFO	PT, OT, HIP
17	14	M	172	54	18.3	3	Tetra	AFO	PT, OT
18	10	M	146	43	20.7	3	Tetra	AFO	PT, HeP
19	11	M	153	47	20.3	3	Tetra	AFO	PT, OT
20	11	M	146	36	16.9	2	Tetra	AFO	PT
21	15	M	160	65	25.4	3	Tetra	None	PT
22 ^DO^	17	F	155	55	22.9	3	Tetra	None	PT
23	14	M	170	71	24.6	3	Tetra	Shoes	PT, LO
24	12	M	150	41	18.2	3	Di	AFO	PT, HIP
25 ^DO^	12	F	160	55	21.5	2	Di	AFO	None
26	12	M	155	54	22.5	3	Tetra	AFO	PT, HIP
27 ^DO^	12	M	160	54	21.1	3	Tetra	AFO	PT, OT
28 ^DO^	9	F	140	38	19.4	3	Di	None	PT, OT, HIP
29	10	M	136	25	13.5	3	Di	AFO	PT, OT
30	15	M	175	68	22.2	2	Tetra	AFO	PT, Petö

M: Male; F: Female; AFO: Ankle-foot orthoses; FO: Foot orthoses; L: Left; R: Right; PT: Physiotherapy; OT: Occupational therapy; HIP: Hippotherapy; LO: Speech therapy; Petö: Conductive support according to Petö; HeP: Alternative therapeutic education; Tetra: Tetraparesis; Di: Diparesis; Soles: Insoles; Shoes: Custom-made orthopedic shoes; Barthel: Barthel Index; DO: Dropout.

**Table 4:** Overview of the results of the MANCOVA of and the interaction effects between the groups with the main factor time and the dependent variables (functional assessments).

Measure (Time)	F-Value	*p*-Value	Partial η^2^
10MWT (SSW)	F = (1, 18) 0.85	0.378	0.045
10MWT (max)	F = (1, 18) 2.62	0.123	0.127
6MWT	F = (1, 18) 0.06	0.8	0.004
GMFM (total)	F = (1, 18) 13.12	0.002 *	0.422
GMFM (D + E)	F = (1, 18) 4.59	0.046 *	0.203

10MWT: 10-metre walking test; SSW: Self-selected walking speed; max: maximum walking speed; 6MWT: 6-minute walking test; GMFM: Gross Motor Function Measure; * = significant; *p* < 0.05; Confidence interval: 95%; *n* = 25.

**Table 5:** Overview of MANCOVA results of and interaction effects between groups with the main factor time and dependent variables (pedobarography).

Measure (Time * Group)	F-Value	*p*-Value	Partial η^2^
Total a-p COP-Movement (mm)	F = (1,10) 0.376	0.554	0.036
Total lateral COP-Movement (mm)	F = (1,10) 0.167	0.691	0.016
Area of sway (mm^2^)	F = (1,10) 2.682	0.133	0.211
Maximum pressure (%) L Maximum pressure (%) R Maximum pressure (%) anterior Maximum pressure (%) posterior	F = (1,10) 0.378 F = (1,10) 0.779 F = (1,10) 0.754 F = (1,10) 4.620	0.552 0.398 0.406 0.057	0.036 0.072 0.070 0.316

a-p: anterior-posterior; L: left; R: right; * = significant; *p* < 0.05; Confidence interval: 95%; *n* = 16.

**Table 6:** Results of the pedobarography measurements pre and post intervention.

	Group	N	MV ± SD	MV ± SD
			pre	post
Total a-p COP-Movement (mm)	Intervention	7	5.2 ± 6.6	6.0 ± 4.6
	Control	9	7.3 ± 6.0	8.5 ± 9.0
Total lateral COP-Movement (mm)	Intervention	7	4.8 ± 4.6	5.2 ± 3.9
	Control	9	11.1 ± 7.9	11.8 ± 11.2
Area of sway (mm^2^)	Intervention	7	6.9 ± 12.0	21.9 ± 45.6
	Control	9	9.2 ± 14.1	22.3 ± 24.9
Maximum pressure (%) L	Intervention	7	50.5 ± 9.7	53.1 ± 12.2
	Control	9	48.8 ± 12.5	49.1 ± 11.0
Maximum pressure (%) R	Intervention	7	49.5 ± 9.7	48.4 ± 11.4
	Control	9	51.2 ± 12.5	49.9 ± 9.9
Maximum pressure (%) anterior	Intervention	7	44.5 ± 4.3	52.3 ± 14.5
	Control	9	51.1 ± 6.0	49.4 ± 7.4
Maximum pressure (%) posterior	Intervention	7	55.5 ± 4.3	51.9 ± 6.4
	Control	9	48.9 ± 6.0	50.6 ± 7.4

MV: Mean value; SD: Standard deviation; Control: Control group; Intervention: Intervention group; a-p: Anterior-posterior; L: Left; R: Right; Confidence interval: 95%; *n* = 16.

**Table 7:** Overview of the MANCOVA results.

Measure	F-Value	*p*-Value	Partial η^2^
Hip L	F = (1,18) 0.527	0.477	0.028
Flexion	F = (1,18) 3.176	0.092	0.15
Extension	F = (1,18) 0.018	0.894	0.001
Internal Rotation	F = (1,18) 1.045	0.32	0.055
External Rotation	F = (1,18) 0.002	0.969	0
Abduction			
Knee L	F = (1,18) 0.004	0.95	0
Flexion	F = (1,18) 0.004	0.951	0
Extension			
Hip R	F = (1,18) 4.423	0.05	0.197
Flexion	F = (1,18) 3.425	0.081	0.16
Extension	F = (1,18) 0.267	0.612	0.015
Internal Rotation	F = (1,18) 1.539	0.231	0.079
External Rotation	F = (1,18) 0.70	0.412	0.038
Abduction			
Knee R	F = (1,18) 1.299	0.269	0.067
Flexion	F = (1,18) 0.485	0.495	0.026
Extension			

Inner-subject contrasts in the comparison between the groups with the main factor time. Included co-variates: age, sex, weight, height and BMI. L: left; R: right; *p* < 0.05; Confidence interval: 95%; *n* = 25.

**Table 8:** Results of the measurement of the passive range of motion of the lower extremities.

	Group	N	MV ± SD	MV ± SD	MV ± SD	MV ± SD
			pre	post	pre	post
			Hip left		Hip right	
Flexion	Intervention	13	102.3 ± 17.5	105.8 ± 15.8	105.4 ± 16.8	106.5 ± 15.7
	Control	12	100.0 ± 10.2	103.3 ± 11.1	101.3 ± 9.1	105.8 ± 8.7
Extension	Intervention	13	−3.5 ± 8.8	0.0 ± 7.4	−3.8 ± 8.5	1.2 ± 5.8
	Control	12	−0.4 ± 8.9	0.8 ± 8.7	−0.4 ± 5.4	1.7 ± 4.4
Internal Rotation	Intervention	13	25.0 ± 18.1	26.9 ± 16.4	21.9 ± 13.5	26.5 ± 13.3
	Control	12	35.8 ± 15.5	37.5 ± 18.8	36.3 ± 18.0	37.9 ± 18.4
External Rotation	Intervention	13	41.5 ± 19.0	48.1 ± 16.9	41.5 ± 18.1	45.4 ± 16.6
	Control	12	45.8 ± 20.5	44.6 ± 21.5	48.8 ± 21.1	47.1 ± 20.1
Abduction	Intervention	13	19.6 ± 8.0	23.1 ± 9.0	15.4 ± 7.5	21.2 ± 9.4
	Control	12	21.3 ± 7.1	23.8 ± 8.8	22.5 ± 10.1	24.2 ± 11.0
			Knee left		Knee right	
Flexion	Intervention	13	130.8 ± 17.7	134.6 ± 16.8	129.6 ± 18.8	131.9 ± 16.5
	Control	12	135.4 ± 18.0	137.9 ± 15.1	136.7 ± 15.7	137.1 ± 15.9
Extension	Intervention	13	−6.2 ± 9.4	−4.2 ± 8.6	−6.5 ± 7.7	−4.6 ± 8.8
	Control	12	−2.9 ± 4.0	−1.3 ± 2.3	−4.6 ± 6.2	−0.8 ± 2.9

MV: Mean value; SD: Standard deviation; Control: Control group; Intervention: Intervention group; Confidence interval: 95%; *n* = 25.

## Data Availability

The data that support the findings of this study are available on request from the corresponding author. The data are not publicly available due to privacy or ethical restrictions.

## Appendix A

**Table A1:** List of interventions carried out in the inpatient therapy concept according to the Template for Intervention Description and Replication (TIDieR) Checklist and Guide.

Intervention	Why?	Who?	How Much?
DocManMed	Treatment of local movement disorders; pain modulation; education of patients and attendants	Doctor with postgraduate training in manualmedicine	4×/week, 30 min
PhysioNeuro	Facilitation of movements and movement transitions; education; improvement of coordination; promotion of independent and directed movements; increase in concentration and reception skills;promotion of participation.	Physiotherapist with postgraduate training in physiotherapy on a neurophysiological basis	5×/week, 30 min
PhysioMT	Treatment of local movement disorders; education; pain modulation	Physiotherapist with postgraduate training in musculoskeletal therapy	
MET	Development of functional strength; training control; coordination training; promotion of independent and directed movements;promotion of participation.	Physiotherapist with postgraduate training in medical exercise therapy	5×/week, 30 min
MassTher	Regulation of muscle tone; pain modulation; physical and mental relaxation; local blood circulation stimulation	Massage therapist	5×/week, 30 min
HAL	Facilitation of movement transitions; coordination and harmonization of standing leg to swing leg phase transition; postural alignment; contextual gait training; support and completion of movementto promote proprioception.	Specially trained physiotherapist	5×/week, 30 min

PhysioNeuro: Physiotherapy on a neurophysiological basis, PhysioMT: Musculoskeletal Therapy (Manual therapy); DocManMed: Physician-performed manual medicine; MET: Medical exercise training; HAL: Hybrid Assistive Limb; MassTher: Massage therapy. All interventions were performed face-to-face at the KMT Hamm during an 11-day inpatient hospital stay.
